# A Dedicated Postpartum Intrauterine Device Inserter: Pilot Experience and Proof of Concept

**DOI:** 10.9745/GHSP-D-15-00355

**Published:** 2016-03-25

**Authors:** Sharad Singh, Vinita Das, Anjoo Agarwal, Rupali Dewan, Pratima Mittal, Renita Bhamrah, Klaira Lerma, Paul D Blumenthal

**Affiliations:** a Population Services International-India, New Delhi, India; b King George Medical University, Queen Mary Hospital, Department of Obstetrics and Gynecology, Lucknow, Uttar Pradesh, India; c Safdarjung Hospital, Department of Obstetrics and Gynecology, New Delhi, India; d Stanford University School of Medicine, Department of Obstetrics and Gynecology, Stanford, CA, USA; e Population Services International, Washington, DC, USA

## Abstract

**Objective:**

To assess the feasibility, acceptability, and safety of a dedicated postpartum intrauterine device (PPIUD) inserter specifically designed for the post-delivery setting. Primary objectives of fundal placement and expulsion rates were assessed. Secondary objectives were participant satisfaction and IUD retention.

**Methods:**

In this pilot proof of concept, we enrolled 80 women who presented for PPIUD insertion at 2 government hospitals in Delhi and Lucknow, India, between March and July 2015. PPIUD insertion was completed with the dedicated inserter in all cases, by trained providers with no prior experience in PPIUD insertion, followed immediately by ultrasound to assess location and fundal placement of the IUD. Follow-up took place at 6 to 8 weeks post-insertion, and ultrasound was used to assess IUD location. Providers and participants also completed satisfaction surveys.

**Results:**

High fundal placement (≤10 mm from uterine fundus) was achieved with the dedicated PPIUD inserter in 82% of cases (n = 65). There were no perforations or infections among the participants and no other complications associated with use of the dedicated inserter. The mean distance between the IUD and the endometrial verge immediately post-insertion was 5.8 mm (range, 0–31; N = 80); this distance at follow-up was also 5.8 mm (range, 0–25; n = 50). Complete expulsion was observed in 6 cases (7.5%), and asymptomatic partial expulsion in 8 cases (10%). Providers reported the majority (93%, n = 74) of insertions to be easy. The majority (74%, n = 59) of participants reported the same level of pain before and after insertion.

**Conclusions:**

This dedicated PPIUD inserter performed as intended and was found to be safe, with high acceptability among the participants and providers. Further study and use of the dedicated inserter may reveal reduced risk of infection among PPIUD users as well as increased convenience compared with standard PPIUD insertion techniques, and could improve acceptability of postpartum IUD provision among providers. The success of this study has led to the initiation of a formal randomized controlled trial in India to further investigate the acceptability of the dedicated inserter.

## Abstract (precis)

Use of the inserter was found to be safe, with high fundal placement in 82% of cases. Complete expulsion occurred in 7.5% of cases and partial expulsion was detected in 10%, comparable with rates in other studies using standard IUD insertion techniques. Further study and use of the dedicated inserter may reveal increased convenience and reduced risk of infection among users and could improve acceptability of postpartum IUD provision among providers.

## INTRODUCTION

In the immediate postpartum time period, there is an opportunity to provide women with contraception they may not otherwise obtain. Data demonstrate a global disparity in family planning services among women in the first year following delivery, with little improvement between 2001 and 2015.^1^^,^^2^ Improving access to long-acting reversible contraceptives (LARCs) immediately postpartum has the potential to improve this gap. Short birth-to-pregnancy intervals (≤18 months) are associated with poor perinatal and maternal health outcomes,^3^^-^^6^ so women and their children may benefit from improved access to immediate postpartum contraception, particularly to LARCs such as intrauterine devices (IUDs).

Post-placental (performed within 10 minutes of placental delivery or while the woman is still in the delivery room) and immediate postpartum (within 48 hours post-delivery) insertions of the IUD are associated with more participant benefits than interval insertion (performed at 6 weeks or more postpartum). Participants report less discomfort and fewer side effects with postpartum IUD (PPIUD) insertion.^7^ In addition, PPIUD insertions are convenient for the participant, provider, and health care system, as they reduce the need for an additional post-discharge family planning visit.

The literature documents higher expulsion rates associated with PPIUD insertions than with interval insertions, but high fundal IUD placement reduces the expulsion rate.^8^ PPIUD expulsion rates appear to be dependent on the skill of the provider in ensuring that the IUD is placed as close to the fundus as possible.^10^ Studies have documented overall low rates of pain, bleeding, infection, and perforation with PPIUD insertion regardless of the timing or insertion technique.^7^^,^^9^

PPIUD insertions are associated with higher expulsion rates than interval insertions, but high fundal IUD placement reduces the expulsion rate.

Until recently, no instrument was specifically designed for IUD insertion in the post-placental or immediate postpartum period. Rather, 2 methods of insertion have emerged for PPIUD insertions: (1) manual insertions, in which the provider removes the IUD from the package and places it on his/her fingers, before manually placing the IUD at the uterine fundus; and (2) forceps insertions, in which the provider removes the IUD from the package, grasps it with forceps, and then places the IUD at the uterine fundus. Each of these approaches requires the IUD to be manipulated by hand, providing opportunity for contamination, possible subsequent infection, and damage to the IUD. In addition, the IUD string of most copper-containing IUDs is not long enough to be visible after PPIUD insertion, which can create uncertainty about IUD location when the woman presents for care later.^11^

Standard PPIUD insertion techniques require providers to manipulate the IUD by hand, providing opportunity for contamination and possible subsequent infection.

Manual insertions have largely been abandoned for reasons related to participant discomfort, possible HIV exposure to the provider, and difficulty of use in morning-after-delivery insertions. Forceps insertions have also become confusing and potentially frustrating to providers, because some types of forceps recommended in global training curricula are not easily available in many countries. The use of forceps also requires special training that can be time consuming and expensive for an insertion technique that is not particularly intuitive.

A dedicated inserter could conceivably increase PPIUD acceptability among both providers and patients if it was convenient and expeditious to use. In addition, preliminary unpublished evidence suggests that the availability of PPIUDs could increase institutional deliveries in rural areas, where maternal mortality is highest and access to contraception the lowest.^12^

**FIGURE 1.**
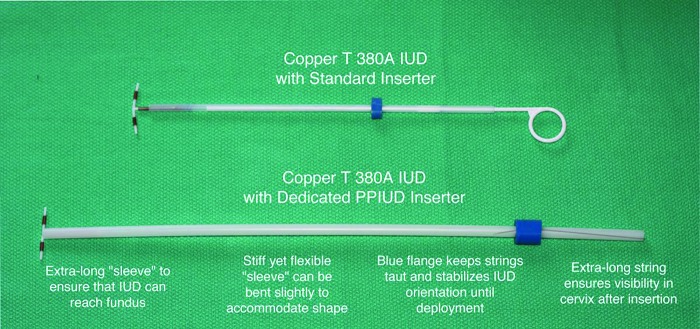
Postpartum Intrauterine Device (PPIUD) Inserter

To address this, a dedicated PPIUD inserter was designed jointly by Population Services International (PSI), The Stanford Program for International Reproductive Education and Services (SPIRES), and Pregna International Ltd. (Figure 1).

The dedicated PPIUD inserter:

- Eliminates the need for specialized instruments such as forceps and allows for a standardized, easy-to-learn technique that mimics interval insertion
- Is made from stiff yet still flexible Silastic that can accommodate the shape of the postpartum uterus
- Comes preloaded in the insertion sleeve—ready to insert—eliminating the need for manipulation and reducing the opportunity for contamination and infection
- Does not require the provider to put his/her hand in the woman’s vagina to insert the IUD, further reducing risk of infection and discomfort
- Has a longer insertion sleeve than the standard IUD inserter to ensure the IUD reaches the fundus easily, further reducing the risk of expulsion and facilitating insertion
- Has a longer string than the standard IUD that is visible following postpartum insertion
- As a dedicated product, could improve acceptability among providers of postpartum IUD provision

A dedicated PPIUD inserter comes preloaded in the insertion sleeve, eliminating the need for manipulation.

A proof-of-concept study was conducted to determine if this new PPIUD inserter, specifically designed for the post-delivery setting, achieves the primary objectives of fundal placement and acceptable expulsion rates, provider and participant acceptability, and feasibility. Secondary objectives of participant satisfaction and IUD retention were also studied.

## METHODS

Between March 2015 and July 2015, we enrolled a convenience sample of women 18 years or older requesting a post-placental or immediate postpartum IUD during prenatal care, at time of delivery, or before 48 hours postpartum at 2 public-sector, government hospitals in Delhi and Lucknow, India. All participants received counseling that included the full range of safe contraceptive methods for postpartum women and the health benefits of spacing pregnancies. Eligible participants met World Health Organization (WHO) medical eligibility criteria for initiating an IUD.^13^ Participants with ruptured membranes more than 18 hours prior to delivery, diagnosis of chorioamnionitis at the time of delivery, unresolved postpartum hemorrhage, or non-vaginal delivery were excluded from the study.

To reduce bias due to possible previous PPIUD insertion experience, health care providers included in the study had a Bachelor of Medicine and Bachelor of Surgery (MBBS) and/or postgraduate degree in obstetrics and gynecology with no prior experience in PPIUD insertion. Study personnel then trained these providers on insertion with the dedicated PPIUD inserter. Training involved a combination of didactic learning, model-based training (with the Mama-U postpartum uterus model developed by Laerdal Global based in Stavanger, Norway, and the specially adapted Noelle maternal and neonatal birthing simulator developed by Gaumard based in Miami, Florida, USA), and supervised clinical practice, lasting a total of no more than 3 days. To view a video demonstrating the models and the PPPIUD insertion technique, see https://www.youtube.com/watch?v = uMcTsuf8XxQ.

Following delivery, participants were assessed for exclusion criteria and if not eligible were withdrawn from the study. Excluded participants were counseled about appropriate family planning methods and were provided with their chosen method by their health care provider.

### Data Collection

Prior to PPIUD insertion, participants were asked to report their perceived pain on a 3-point scale of “no pain,” “bearable,” and “unbearable.” The participant’s IUD was placed using the dedicated PPIUD inserter up to 48 hours post-delivery, but as close to delivery as possible. The timing of insertion with respect to delivery was recorded. An abdominal ultrasound was performed immediately post-insertion to assess fundal placement of the IUD. The distance between the endometrial verge (the fundal termination of the endometrium) and the uppermost (leading) aspect of the IUD was measured. In addition, the distance between the external cervical os and the uterine fundus was assessed with the inserter itself, which had markings to indicated uterine depth.

Immediately post-insertion, participants were asked to report their perceived pain on the same 3-point scale of “no pain,” “bearable,” and “unbearable.” Prior to discharge, participants completed a questionnaire with a counselor that was designed to capture participant satisfaction, pain experienced, counseling provided, experience at hospital, and if they would recommend PPIUD insertion to a friend or family member. Additionally, the health care provider inserting the IUD using the dedicated inserter completed a satisfaction questionnaire. This questionnaire assessed the ease of insertion on a 3-point scale (“easy,” “slightly difficult,” “difficult”), reinsertions (if any), and location of the IUD on ultrasound.

Participants were contacted by telephone every week after PPIUD insertion for health-related information and to remind the participant of the importance of attending the follow-up visit 6 to 8 weeks post-insertion. In case of any complaint of discomfort or expulsion, the participant was encouraged to visit the hospital immediately.

At the follow-up visit, up to 8 weeks post-insertion, an ultrasound was performed to assess the position of the IUD with the same IUD-endometrial verge distance being recorded. If a complete expulsion (complete spontaneous expulsion of the IUD from the uterus) or a partial expulsion (asymptomatic descent of the IUD such that it could be seen in the cervix at examination) was noted, this was recorded in the case reporting form. Follow-up visits were performed at the 2 hospitals where insertions took place as well as at additional, secondary clinical sites. Participants were also asked at the follow-up visit to indicate their satisfaction with their experience and whether they would recommend PPIUD insertion to a friend or family member.

### Data Analysis

Data were collected on paper forms and entered into Excel spreadsheets. Statistical analyses were performed with SPSS version 23 and SYSTAT version 13. Sociodemographic characteristics of the participants were analyzed using descriptive statistics. Student’s *t* test was used to compare means, and non-parametric tests were used to compare medians.

### Ethics Approval

Study approval was obtained from the Drug Controller General of India (DCGI) as well as the Ethics Committees of the relevant hospitals. The study was registered with the Clinical Trial Registry of India (CTRI). All participants provided informed consent.

## RESULTS

### Background Characteristics

Study personnel trained 11 health care providers on how to use the dedicated PPIUD inserter. During the study period, 80 participants provided consent and were enrolled (Table 1). Women ranged from 18 years of age to 37 years. All participants had at least 1 living child, and parity ranged from 1 to 6. The majority (75%) of participants delivered at or after 37 weeks gestation. Nearly all women had either a normal vaginal delivery with episiotomy (59%, n = 47) or a normal vaginal delivery without episiotomy (39%, n = 31); 2 participants (2%) had an assisted vaginal delivery with either vacuum or forceps.

**TABLE 1:** Baseline Characteristics of Study Participants in India (N* = *80)

	No. (%)
Age, years	
18–20	8 (10)
21–30	65 (81)
>30	7 (9)
No. of living children	
1	23 (29)
2	35 (44)
≥3	22 (27)
Gestation at delivery, weeks	
32–37	20 (25)
>37	60 (75)
Timing of contraceptive counseling	
Prenatal	8 (10)
Early labor	47 (59)
Postpartum (up to 48 hours post-delivery)	25 (31)
Duration of membrane rupture, hours	
<6	66 (83)
6–12	13 (16)
>12	1 (1)
Type of delivery	
Normal vaginal	31 (39)
Normal vaginal with episiotomy	47 (59)
Assisted vaginal (vacuum, forceps)	2 (2)
Time interval between delivery and PPIUD insertion, hours	
<1	49 (61)
1–6	26 (33)
>6	5 (6)

### Timing of PPIUD Insertion

The majority of participants in the study received contraceptive counseling for their PPIUD while in early or prodromal labor (59%); no counseling took place during active labor. Most (61%, n = 49) participants received their PPIUD insertion less than 1 hour after vaginal delivery, while 33% (n = 26) received their PPIUD insertion between 1 and 6 hours after vaginal delivery and 6% (n = 5) more than 6 hours after vaginal delivery (Table 1). The overall median time difference between the time of delivery and the time of insertion was 42 minutes, and the mode was 15 minutes.

### Fundal Placement of IUD

Fundal placement (≤10 mm from the fundus) with the PPIUD inserter was achieved in 82% of cases (n = 65) (Table 2). Average fundo-cervical length was 17.5 cm, meaning that over 80% of the participants had their IUD inserted within 10 mm of the fundus or approximately 95% of the distance to the fundus from the cervix. Removal and reinsertion was performed in 3 participants, as the provider was initially not sure of fundal placement. No perforations were reported or observed on ultrasound, and no infections occurred among the participants. In addition, there were no other complications associated with use of the dedicated PPIUD inserter.

Fundal placement with the PPIUD inserter was achieved in 82% of the cases.

**TABLE 2:** Key Performance Results of Dedicated PPIUD Inserter Among Postpartum Women in India (N = 80)

	No. (%)
Distance from fundus to IUD on ultrasound (immediately post-insertion), mm	
<6	58 (72.5)
6–10	7 (8.8)
11–20	10 (12.5)
>20	5 (6.3)
Change in participant pain status after insertion	
Same	59 (73.8)
Increased	7 (8.8)
Decreased	14 (17.5)
Provider reported ease of PPIUD insertion	
Easy	74 (92.5)
Slightly difficult	2 (2.5)
Difficult	4 (5.0)
IUD location at follow-up	
Retention	61 (76.3)
Partial expulsion	8 (10.0)
Complete expulsion	6^a^ (7.5)
Removal	5^a^ (6.3)

a One case occurred during an episode of delayed postpartum hemorrhage.

### Women’s Reports of Pain and Providers’ Reports of Ease of Insertion

The participant satisfaction questionnaire revealed that 74% of participants (n = 59) experienced the same level of pain before and after PPIUD insertion, 17% (n = 14) reported a decrease in pain compared with just before insertion, and only 9% (n = 7) reported an increase of pain. Health care providers reported the vast majority (93%, n = 74) of insertions to be easy (Table 2).

### Expulsion Rates

All 80 participants completed follow-up, defined as a clinical visit in which the presence or expulsion of the IUD was verified. Of the 50 participants who had ultrasound at follow-up, the mean distance measured with ultrasound from the endometrial verge to the IUD was 5.8 mm (standard deviation, 6; range, 0–25) (Table 3).

**TABLE 3:** Distance (mm) From Fundus to IUD Immediately Post-Insertion and at Follow-Up

	Mean (SD; Range)
Immediately post-insertion (N = 80)	5.8 (7; 0-31)
At follow-up (n = 50)	5.8 (6; 0-25)

Abbreviation: SD, standard deviation.

**FIGURE 2.**
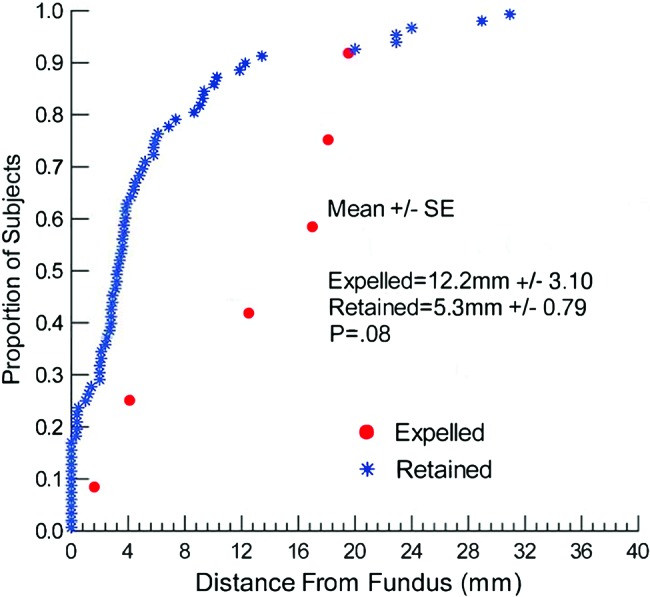
IUD Distance From Fundus by Expulsion Status (N=80) Abbreviation: SE, standard error.

**FIGURE 3.**
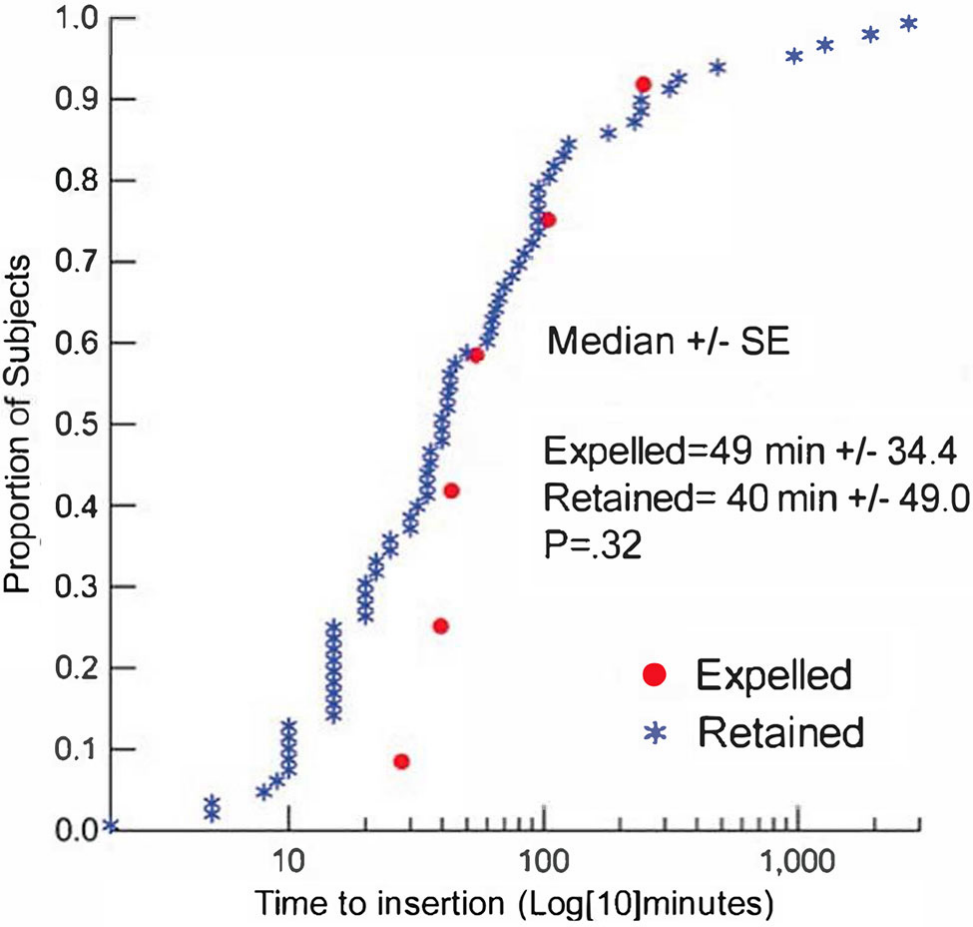
Time to PPIUD Insertion After Delivery by Expulsion Status (N=80) Abbreviation: SE, standard error.

Among all 80 participants, by the follow-up visit, the IUD was completely expelled in only 6 cases (7.5%), partially expelled in 8 cases (10.0%), and removed in 5 cases (6.3%) for social and clinical reasons (Table 2). The mean distance of the IUD from the fundus (on immediate post-insertion ultrasound) among those cases with an expelled IUD at the follow-up visit was 12.2 mm compared with 5.3 mm among women whose IUD was retained at the follow-up visit (*P* = .08) (Figure 2). The median time from delivery to insertion was 49 minutes among women with an expelled IUD compared with 40 minutes among women who retained their IUDs (*P* = .32) (Figure 3).

Complete expulsion occurred in 6 cases (7.5%).

Of the 4 insertions reported by providers to be difficult, 3 resulted in complete expulsion and 1 in a partial expulsion. Interestingly, the mean distance from the endometrial verge to the IUD (on immediate post-insertion ultrasound) among the cases reported as difficult was 16 mm (range, 5–31) compared with 5.8 mm for the total group (*P* = .16). Among difficult insertions, the median time from delivery to insertion was 65 minutes compared with 41 minutes among insertions assessed as easy or slightly difficult.

In the 6 cases of complete IUD expulsion, 2 were expelled on the same day as insertion (1 of which occurred during an episode of delayed postpartum hemorrhage). In the 5 reported cases of IUD removal, 2 participants removed the IUD themselves by pulling the strings and 2 participants had their IUD removed by a private health care provider (speculatively due to psychosocial motivations). The reasons for removal in the remaining case are uncertain.

### Client Satisfaction

After insertion, 100% of the participants stated that their provider met or exceeded their expectations, and 99% of participants reported the overall experience met or exceeded their expectations. Participants’ experience at the hospital was reported as better than expected in 38% of cases and the same as expected in 61%. Almost all participants said they were satisfied with counseling and the decision to get a PPIUD and would recommend this method of contraception to their friends and family members.

## DISCUSSION

In this proof-of-concept study, the dedicated PPIUD inserter functioned well and very much as anticipated, with over 80% of IUDs being placed high (≤10mm from the IUD to the fundus internally) in the uterine fundus. In this very carefully followed group, complete IUD expulsion at up to 8 weeks post-insertion was observed in 6 (7.5%) of the 80 participants and partial expulsions reported in 8 (10.0%). These rates are consonant with the published literature,^7^^,^^9^ especially in studies with high rates of clinical follow-up.^14^^,^^15^

Expulsion rates with the dedicated PPIUD inserter were comparable with rates in other studies using standard IUD insertion techniques.

Evidence suggests that placement of the IUD at the fundus and the skill of the provider are important factors for effective PPIUD service delivery.^16^ Data have also indicated that PPIUD insertions done within 10 minutes (post-placental) of delivery result in lower expulsion rates than those done after 2 hours.^7^^,^^10^^,^^17^ However, in many of these studies, high fundal placement was not mentioned or documented as a primary insertion objective. The lower uterine segment contracts and assumes its non-gravid curvature starting within 24 hours post-delivery. Thus, unless a concerted effort was made to achieve fundal placement, it may have been potentially less likely and expulsion rates possibly higher when insertions were done later rather than immediately post-delivery.^10^ Our data, albeit limited, but derived from a study in which fundal placement was a definite objective, do not show an effect of timing on subsequent expulsion. Thus, PPIUD expulsion rates appear to be mainly dependent on the skill of the provider in ensuring that the IUD is placed as high as possible to the fundus.

PPIUD expulsion rates seem to be dependent on both the timing of the insertion and the skill of the provider in ensuring proper fundal placement.

Since fundal placement is held to be an important factor for increasing IUD retention, techniques that can realize such placement may reduce the expulsion rate and enhance overall service delivery. Again, with respect to this criterion, the inserter being assessed here functioned well. Given the reduced diameter of the dedicated inserter, compared with forceps, and the fewer intrauterine manipulations involved, the dedicated inserter may be both more convenient and comfortable than forceps insertions for women having an insertion after they have left the delivery room.

Provider experience also affects the rate of expulsion, most likely as a function of proper IUD placement. In the study by Chi et al. (1989), providers with experience in vaginal PPIUD insertion had a 6.9% expulsion rate whereas inexperienced providers had a 12% rate of expulsion.^17^ In the data reported here, when the provider reported the insertion to be “difficult” (a subgroup that contained 3 of the 6 expulsions in the study), there was a trend toward later insertion and a greater distance from the fundus after insertion.

Importantly, our study documented 100% follow-up up to 8 weeks post-insertion, and it is noteworthy that there were no unrecognized expulsions during that time. A study by Bednarek et al. (2011) reported follow-up rates of 73%, which may have resulted in an underestimate of rates of expulsion, unintended pregnancy, and infection.^18^ In an upcoming planned randomized controlled trial (RCT), with a larger number of participants, we hope to establish associations between fundal placement and expulsion rates and between expulsion and time between delivery and IUD placement.

As the literature on IUD expulsion rates matures, a pattern of expected “market rates” of expulsions may be emerging:

- 0%–5% after interval insertions^19^^-^^21^
- 3%–5% after 1st trimester abortions^18^
- 4%–8% after 2nd trimester abortions^22^
- 8% after cesarean delivery^23^
- 5%–25% after a term vaginal delivery^7^^,^^9^

Such rates make sense in terms of the physiologic and pathologic states relating to uterine size, propulsive forces in the uterus acting on the IUD, cervical dilation, and blood flow. This study’s 7.5% rate for complete, spontaneous expulsion is very consistent with the published peer-reviewed literature on this topic. With high rates of follow-up in this study compared with many other studies in this topic area, the “partial expulsions” might not have been captured in other, less rigorous studies. It is important to note that almost 90% of women in this study continued to have an IUD that was conveniently obtained, and a method that studies indicate might not have been provided to the woman at all had it not been provided immediately postpartum.^23^^,^^24^ As Blumenthal and Goldthwaite state, “a woman simply cannot continue to use an IUD that she never got.”^24^

As stated above, there were no unrecognized expulsions in this study. Expulsions are neither dangerous nor painful for women; when expulsion is noted, another IUD can easily be inserted or another contraceptive method obtained. *It is unrecognized expulsions that leave the woman exposed to unintended pregnancy, and none of those occurred in this study.* Finally, Salcedo et al. (2013) indicate that, depending on the cost of the IUD and the service provided, expulsion rates can be as high as 30% and the IUD still remains a cost-effective approach.^25^

There were no unrecognized expulsions in this study.

### Limitations

This study was not without limitations. Counseling and enrolling women in the prenatal period for the study was difficult or not possible; some women who were counseled in prenatal care were excluded because of cesarean delivery or because of delivery at other birthing centers. The majority of participants enrolled for the study were counseled during the early labor and postpartum periods. Ensuring follow-up was also not without challenge in India, as many women traditionally visit a maternity home post-delivery. Due to this tradition, many women were unable to come to their respective study site (i.e., where the woman delivered and the IUD was inserted) for follow-up, so the study protocol allowed the woman to visit a secondary study site for follow-up. At these secondary sites, data on retention or expulsion were obtained, but ultrasound was not always possible. We also acknowledge the limitation of the small sample size in this study and plan to address this in future studies.

In addition, although price of the inserter was not considered in this pilot, the manufacturer indicates that when used in programs, the total cost of the inserter (which includes the IUD itself) would be less than US$1, thus making this a very cost-effective device, and one that compares well with other methods in common use. As always, the cost of actually getting an IUD at the time of the delivery (given that no other instruments are necessary for insertion) must be considered in comparison with the *intended* use of forceps insertion but which may not occur due to the absence of the required instrument.

The inclusion criteria for health care providers to be naïve to PPIUD insertion was an additional limitation of the study. The current guidelines of the state government in India are for doctors to be trained in PPIUD insertion immediately upon joining a government hospital. Thus, it was difficult to find doctors truly inexperienced in PPIUD insertion. In addition, due to the multiple responsibilities imposed on doctors at an early stage of training, it was not always possible for them to be present in the labor and delivery area when an insertion needed to be performed. As a result, the majority of insertions at 1 study site were done by 1 provider (31 of 50 insertions).

## CONCLUSION

Overall, this dedicated PPIUD inserter was found to be safe and effective with high acceptability among the participants and providers. The inserter performed well and as anticipated, and there were no adverse events or complications associated with its use. Complete IUD expulsion up to 8 weeks post-insertion was observed in 7.5% of cases. As a proof-of-concept study, it was not powered to perform inferential statistics for analyzing associations between variables. The success of this study has led to the initiation of a formal RCT in India to further investigate the acceptability of the dedicated PPIUD inserter.

## Notes

**Peer Reviewed**
